# Functional analysis of RIP toxins from the *Drosophila* endosymbiont *Spiroplasma poulsonii*

**DOI:** 10.1186/s12866-019-1410-1

**Published:** 2019-02-20

**Authors:** Mario Gonzalo Garcia-Arraez, Florent Masson, Juan Camilo Paredes Escobar, Bruno Lemaitre

**Affiliations:** 1 Global Health Institute, School of Life Science, École Polytechnique Fédérale de Lausanne (EPFL), Lausanne, Switzerland; 2 International Centre of Insect Physiology and Ecology (ICIPE), Kasarani, Nairobi, Kenya

**Keywords:** *Spiroplasma*, Endosymbiosis, Ribosome inactivating protein, *Drosophila*

## Abstract

**Background:**

Insects frequently live in close relationship with symbiotic bacteria that carry out beneficial functions for their host, like protection against parasites and viruses. However, in some cases, the mutualistic nature of such associations is put into question because of detrimental phenotypes caused by the symbiont. One example is the association between the vertically transmitted facultative endosymbiont *Spiroplasma poulsonii* and its natural host *Drosophila melanogaster.* Whereas *S. poulsonii* protects its host against parasitoid wasps and nematodes by the action of toxins from the family of Ribosome Inactivating Proteins (RIPs), the presence of *S. poulsonii* has been reported to reduce host’s life span and to kill male embryos by a toxin called *Spaid*. In this work, we investigate the harmful effects of *Spiroplasma* RIPs on *Drosophila* in the absence of parasite infection.

**Results:**

We show that only two *Spiroplasma* RIPs (*SpRIP1* and *SpRIP2*) among the five RIP genes encoded in the *S. poulsonii* genome are significantly expressed during the whole *Drosophila* life cycle. Heterologous expression of *SpRIP1* and *2* in uninfected flies confirms their toxicity, as indicated by a reduction of *Drosophila* lifespan and hemocyte number. We also show that RIPs can cause the death of some embryos, including females.

**Conclusion:**

Our results indicate that RIPs released by *S. poulsonii* contribute to the reduction of host lifespan and embryo mortality. This suggests that *SpRIPs* may impact the insect-symbiont homeostasis beyond their protective function against parasites.

**Electronic supplementary material:**

The online version of this article (10.1186/s12866-019-1410-1) contains supplementary material, which is available to authorized users.

## Background

Endosymbiosis refers to a persistent interaction between two partners, generally a eukaryotic host and a microbial symbiont that lives within the host’s body. Such interactions are particularly frequent in insects, of which more than half of species are estimated to harbor at least one endosymbiont [1, 2]. Insect endosymbionts can affect their host in multiple ways, including beneficial effects such as metabolic complementation, heat tolerance or protection against viruses and parasites [3–7]. However some endosymbiotic associations can also have detrimental consequences for the insect fitness, such as a decreased lifespan or fertility [8, 9]. Among the most widespread facultative endosymbionts that manipulate insect reproduction are the genera *Wolbachia* and *Spiroplasma* [10]*.*

*Spiroplasma poulsonii* (hereafter *Spiroplasma*) is a natural symbiont of the fruit fly *Drosophila melanogaster* [11–13]*.* It lives extracellularly in the fly hemolymph and is vertically transmitted by trans-ovarial transfer. *Spiroplasma* colonizes the germline during vitellogenesis by co-opting the yolk transport and internalization machinery [14]. Intriguingly, it completely lacks a cell-wall and thus immunogenic surface molecules, such as peptidoglycan, which renders it invisible for the host immune system [15–19]. In adult flies, *Spiroplasma* grows over time reaching a titer of 10^5^-10^6^ bacteria per μl of hemolymph [20]. *Spiroplasma* infection shortens the lifespan of *Drosophila,* suggesting that either the bacteria causes damages only at high titer, or that the damages take time to kill the host [19]. Interestingly, the growth of *S. poulsonii* is limited by the availability of host lipids, preventing its overgrowth in condition of nutrient scarcity [19].

One of the most striking phenotypes caused by *Spiroplasma* is male-killing, whereby infected male embryos die during their development while most infected females survive [12]. As *Spiroplasma* is only transmitted by female flies, male-killing is thought to favor the spread of the bacteria among host natural populations [21]. Recently, a *Spiroplasma* toxin containing Ankyrin-repeats, named *Spiroplasma* Androcidin (Spaid), has been described as a crucial male-killing agent [22, 23]*.* Heterologous expression of *Spaid* in uninfected flies is sufficient to kill males. Moreover, its expression during early embryogenesis induces DNA-damage-dependent apoptosis and defective neurogenesis in uninfected male embryos, which fully recapitulates male-killing phenotypes [24–27].

Studies have shown that in some contexts, *Spiroplasma* can also provide a benefit to its host as they mediate protection against parasitoid wasps and nematodes in several *Drosophila* species [28–31]. Protection is a major ecological benefit that can lead to a fast spreading of *Spiroplasma* in wild populations [32]. Two complementary mechanisms have been implicated in *Spiroplasma* protection against parasites: a metabolic competition for host lipids between *Spiroplasma* and the parasites, and *Spiroplasma* production of Ribosome-Inactivating Proteins (RIPs) that damage ribosomes of both wasp eggs and nematodes [28, 30, 31]. RIPs are found in plants and bacteria, where they act as a defense against eukaryotic parasites [30, 31, 33–35]. They recognize a conserved region of the 28S ribosomal RNA called the Sarcin-Ricin Loop (SRL). The secondary structure of the SRL consists in a hairpin loop displaying an adenine that is necessary for protein synthesis [36]. RIPs cleave the central adenine from the SRL in a process called depurination, thus blocking protein synthesis [37].

In this article we investigated the role of RIPs produced by the facultative endosymbiont *S. poulsonii* (hereafter *Sp*RIPs) in its natural host *D. melanogaster.* Similarly to the reduced lifespan observed in infected flies, we show that heterologous expression of *SpRIPs* coding genes in uninfected flies shortens their life span. Furthermore, uninfected-embryos expressing *SpRIPs* have high mortality rate and a female-biased sex-ratio among the surviving individuals, suggesting that males may be more sensitive to the ectopic expression of this toxin.

## Results

### *Sp*RIPs depurinate the 28S rRNA of *D. melanogaster*

*S. poulsonii* genome contains five genes encoding RIPs (*SpRIP1-5*) [20, 30, 31, 38]. All of them have a signal peptide, suggesting a secretion of the mature protein, and a conserved N-glycosidase domain in charge of the depurination reaction [30, 31]. All copies are chromosomal, suggesting that they are very stable compared to other endosymbiont toxin coding genes that are located on plasmids or mobile elements such as *Spaid* in *S. poulsonii* or the cytoplasmic incompatibility factor of *Wolbachia* [20, 23, 39]. Transcriptome analysis has shown that only two of them, *SpRIP1* and *SpRIP2*, are significantly expressed in vivo and in vitro, pointing to a possible pseudogenization of *SpRIP3*, *4* and *5* [20, 30].

**Fig. 1:**
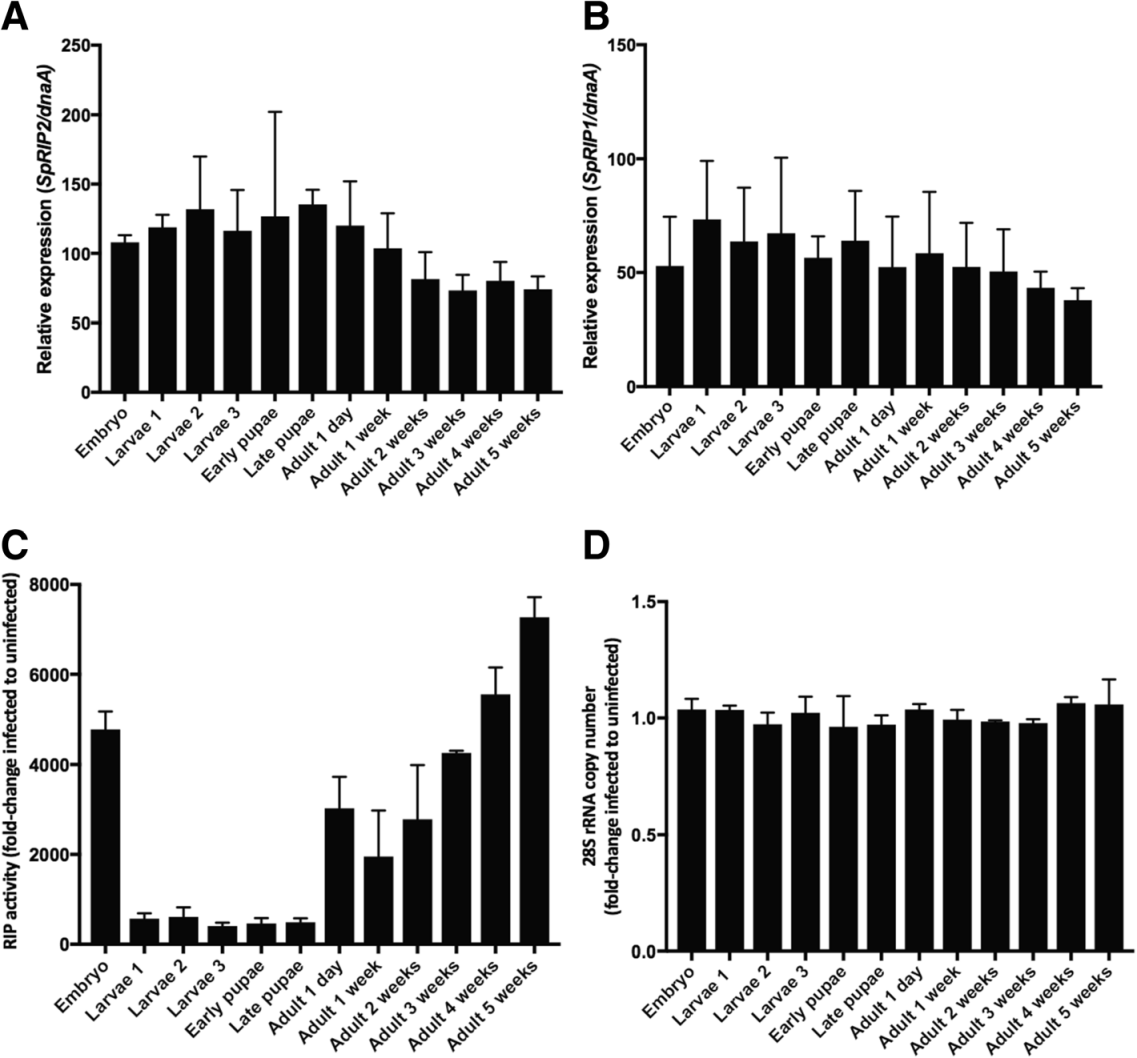
**a** *SpRIP1* expression level in infected flies along *Drosophila* development stages (One way ANOVA; development stage *p* = 0.9055). **b** *SpRIP2* expression level in infected flies along *Drosophila* development stages (One way ANOVA; development stage *p* = 0.5129). **c** RIP activity in infected flies compared to uninfected flies (Two way ANOVA; *Spiroplasma* infection p*** < 0.0001; development stages p*** < 0.0001; interaction p*** < 0.0001). **d** Intact 28S rRNA quantification in infected versus uninfected flies along *Drosophila* development stages

To confirm the expression pattern of *SpRIPs* in infected flies, we performed RT-qPCR analysis on each of the *SpRIPs*. We confirmed that *SpRIP1* and *SpRIP2* are strongly expressed by *S. poulsonii* in *D. melanogaster* with no significant changes in expression level along the fly life cycle (Fig. 1a and b). Very low levels of *SpRIP 3*, *4* and *5* transcripts were detected, in accordance with the literature (Additional file 1: Figure S1). We then measured RIP activity using a RT-qPCR assay. This assay relies on the ability of reverse transcriptases to incorporate a thymine in complementary DNA in place of the void position present on the depurinated RNA molecule. It is then possible to design primers that bind specifically to the intact cDNA (containing an adenine) or to the depurinated one (containing a thymine) [31]. Comparisons between infected and uninfected flies confirmed that *S. poulsonii* depurinates the 28S rRNA of *Drosophila,* as previously shown for larvae and 1 week old adult flies [30]. Monitoring RIP activity along the whole *Drosophila* lifecycle revealed particularly high levels of depurination in embryos and old adult flies (Fig. 1c). A control assay using primers amplifying fragments outside of the SRL showed that the total number of 28 rRNA transcripts was the same between infected and uninfected flies (Fig. 1d). As the level of expression of *SpRIP* in *Spiroplasma* is constant, the high level of depurination in embryos and old adult flies likely results from the higher *Spiroplasma* titer in the host at these stages [19].

### *SpRIP1* and *SpRIP2* expression is toxic for *Drosophila melanogaster*

**Fig. 2:**
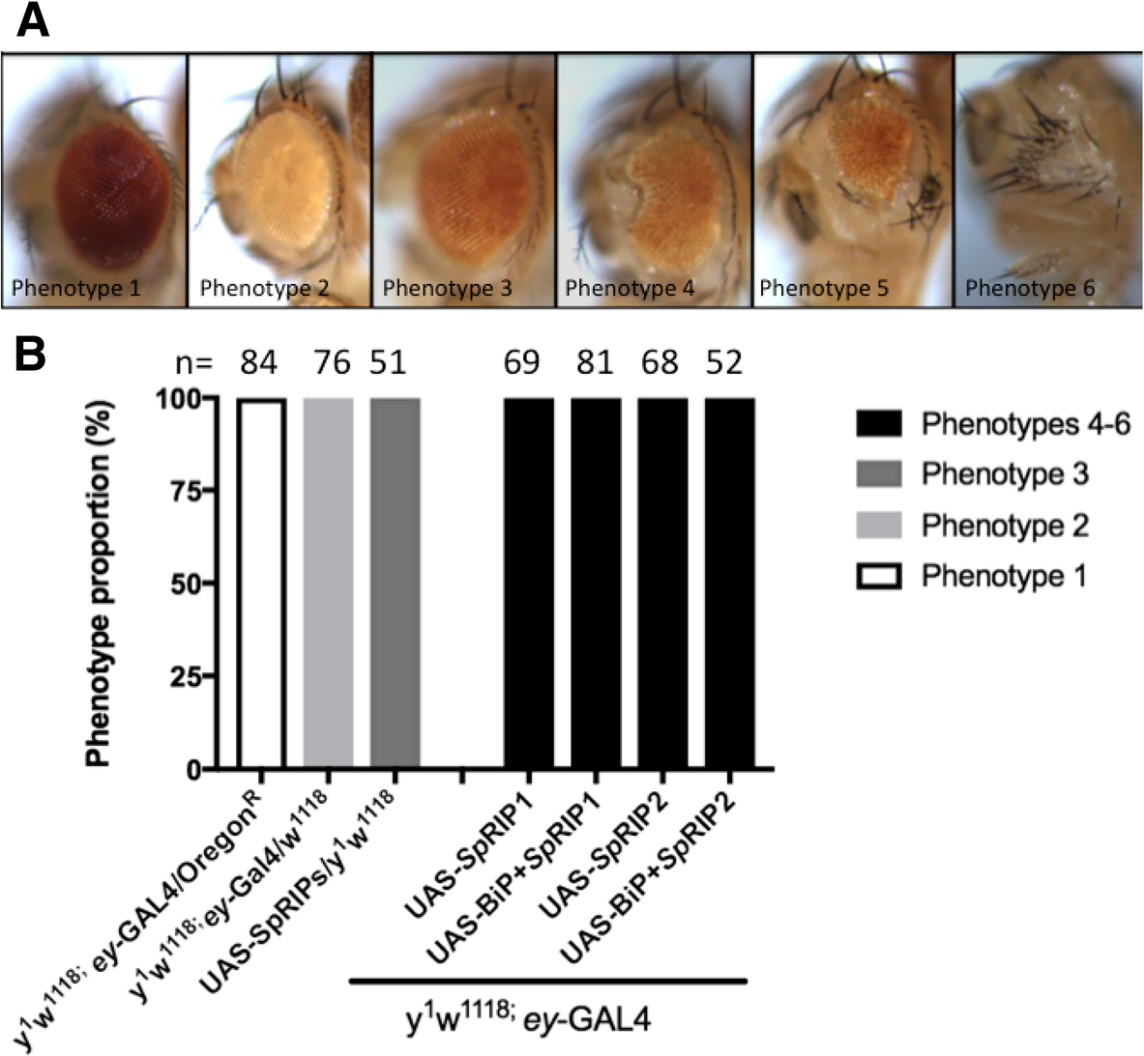
Rough Eye Phenotype assay. **a** Representative bright-field images of *Drosophila* eye phenotypes obtained during the assay. **b** Percentage of each phenotype. n indicates the number of flies obtained for each cross. Each cross has been repeated three independent times

We generated four different transgenic fly lines expressing singly *SpRIP1*, *SpRIP2*, BiP + *SpRIP1* or BiP + *SpRIP2* under the control of the *GAL4/UAS* system [40]. BiP is a signal peptide used to trigger the secretion of proteins in *D. melanogaster* [41, 42]. The toxicity of these constructs was tested using the “Rough Eye Phenotypes” (REP) assay, which allows to study the activity of a putative toxin driven by an eye-specific driver (*ey-GAL4*) to observe eventual deleterious effect of the protein on this organ’s structure [43, 44]. The REP assay allows to study toxin activity by monitoring defects including loss of bristles, fusion of ommatidias, necrosis, loss of pigmentation and reduced eye size [43, 45]. All control flies developed a normal eye structure. On the contrary, flies expressing *UAS-SpRIPs* under *ey-GAL4* control developed a reduced eye along with severe abnormalities, and in some cases no eye at all (Fig. 2). This demonstrates that both *Sp*RIP1 and 2 act as toxins on *Drosophila* cells*.*

### Ectopic expression of *SpRIP1* and *SpRIP2* decreases uninfected flies life span

**Fig. 3:**
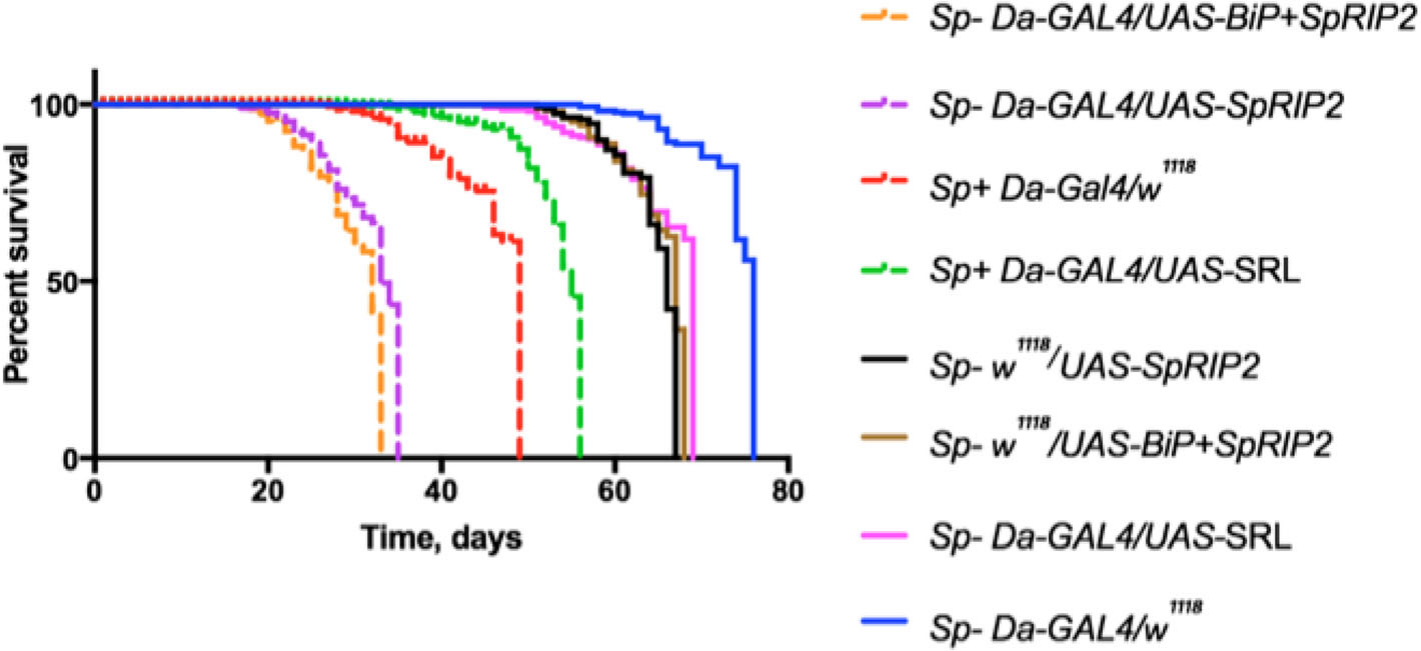
Effect of *SpRIP2* expression on *D. melanogaster* lifespan. *Sp*- and *Sp +* refer to uninfected or *Spiroplasma*-infected condition respectively. *UAS* constructs were driven by the ubiquitous *Da-GAL4* driver. Sample labels are ordered from the shortest to the longest lifespan. Plain lines represent uninfected stocks and controls. Dashed lines represent either infected flies or expressing *SpRIPs*. Pairwise comparison of survival fits where analyzed by Log-rank (Mantel-Cox) test

*Spiroplasma*-infected flies have a shorter lifespan compared to uninfected ones [16]. Moreover, old infected flies have been reported to have a decreased climbing activity which suggests neurological damages [19]. We first confirm this phenotype, observing that infected flies have a lifespan reduced by about 20 days (Fig. 3 and Additional file 2: Figure S2). As *Spiroplasma* resides in the hemolymph, we hypothesized that the impact of *Spiroplasma* on host lifespan could be due to accumulation of a toxin released in the hemolymph. Accordingly, proteomics analysis of hemolymph of 2 weeks old *Spiroplasma*-infected flies revealed the presence of *Sp*RIP1 and *Sp*RIP2 (S. Rommelaere, F. Masson, and B. Lemaitre, unpublished data).

To further address the impact of *Sp*RIPs on *Drosophila* viability, we tested the effect of ectopic expression of *SpRIPs* on the life span of uninfected flies. *Drosophila* expressing *SpRIP1* or *BiP + SpRIP1* constructs did not develop further than larval instars, preventing the use of these constructs for lifespan analysis. Interestingly, uninfected flies expressing *SpRIP2* and *BiP* + *SpRIP2* had a markedly decreased lifespan by about 30 days in average compared to uninfected flies, which live about 75 days (Fig. 3; Logrank test p*** < 0.0001). The lifespan of these transgenic lines was also shorter than the one of infected flies, which live about 45 days (Fig. 3; Logrank test p*** < 0.0001). The lifespans seem to be depending on the expression level and activity of the RIPs (Additional file 3: Figure S3 and Additional file 4: Figure S4).

To further test the implication of *Sp*RIPs in premature adult lethality, we generated a transgenic fly line expressing a 1492 bp fragment of the 28S rRNA under the control of a *UAS* upstream sequence [40]. This fragment contains the conserved SRL targeted by RIPs and was designed to buffer RIP activity by increasing the number of targets for the toxin, thus working as an antidote. *Spiroplasma*-infected flies with ubiquitous expression of SRL fragment display an increase in their lifespan by about 5 days compared to infected wild-type flies (Fig. 3; Logrank test p**** < 0.0001). Collectively, these results are consistent with the implication of *Sp*RIP in shortening *Drosophila* life span.

### *Spiroplasma*-infected flies and uninfected flies expressing *SpRIP2* have reduced hemocyte count

**Fig. 4:**
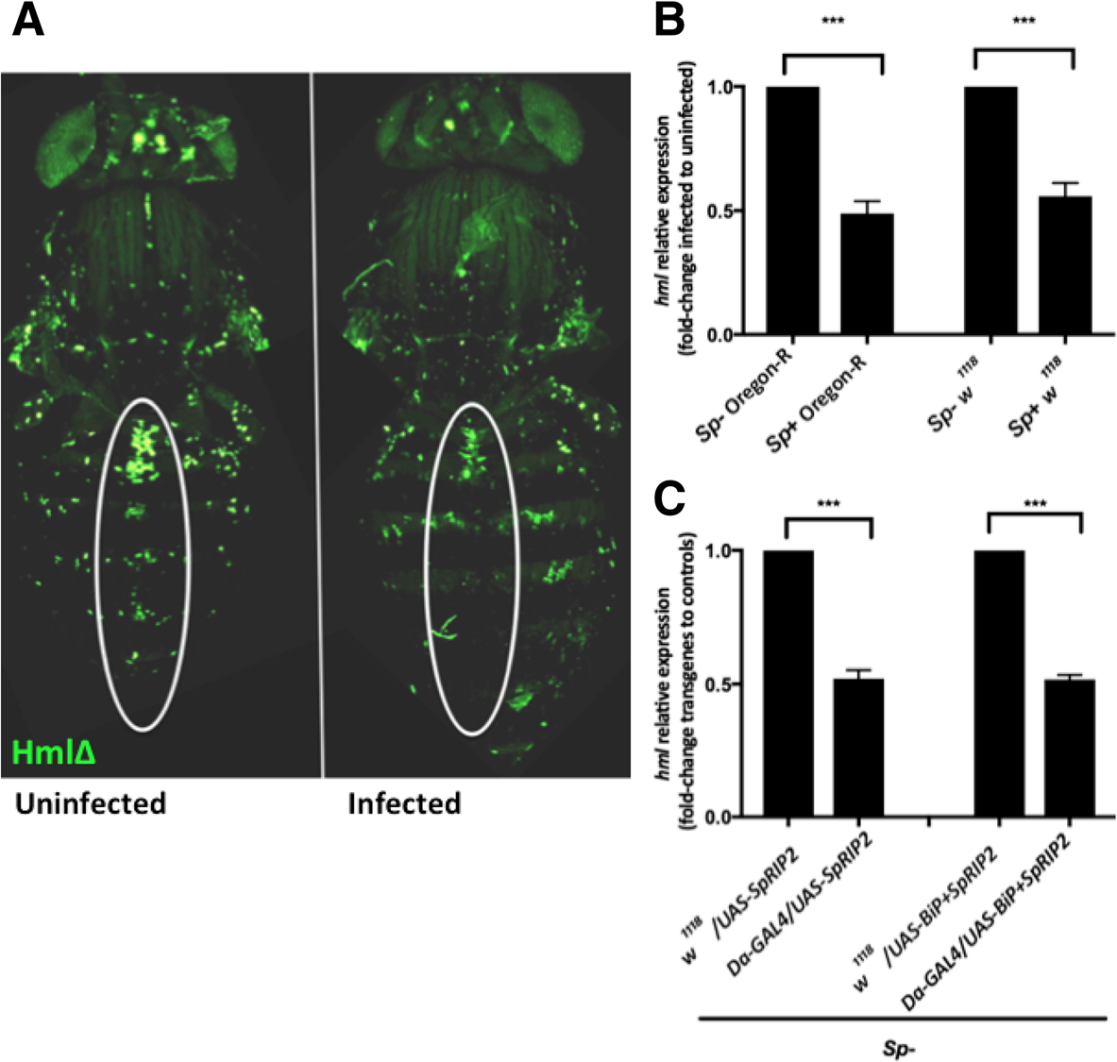
**a** Confocal image of *HmlΔ*-*GAL4/UAS-GFP* adult flies. In uninfected flies, hemocyte patches are mostly located within the white circle, following the antero-posterior axis. In infected flies only a few patches remain and have lower fluorescence intensity. **b** *hml* transcription level in infected and uninfected adult wild type flies (Dunnett’s multiple comparisons test p*** < 0.0001). **c** *hml* transcription level in lines expressing *UAS-SpRIP2* and *UAS-BiP + SpRIP2* under *Da-GAL4* control. Expression of both constructs lead to a decrease in hemocyte number (Dunnett’s multiple comparisons test p*** < 0.0001). *Sp*- and *Sp +* refer to uninfected or *Spiroplasma*-infected condition respectively. Controls are normalized as 1

As *S. poulsonii* is found in the fly hemolymph, we hypothesized that hemocytes should be the most affected cell type by RIP toxins. We thus visualized hemocyte in *Spiroplasma*-infected and uninfected adult flies, carrying the hemocyte marker *Hml-GAL4 > UAS-GFP*. In uninfected adult flies, sessile hemocytes are found in patches beneath the cuticle in the middle of the dorsal abdomen [46]. Interestingly, *Spiroplasma*-infected flies have reduced number of sessile patches (Fig. 4a). To confirm this observation, we indirectly estimated the number of hemocytes in adult flies by monitoring the expression of *hemolectin* (*hml*), a gene which expression is hemocyte-specific. Consistent with a reduction of the number of hemocytes, the expression of *hml* was halved in *Spiroplasma*-infected flies compared to uninfected ones in two different wild type strains (Fig. 4b). We conclude that the presence of *Spiroplasma* greatly reduces the number of hemocytes. This reduction could reflect the shortening of lifespan as a decreased hemocyte count is one of the hallmark of aging in flies [47]. To test whether *Sp*RIPs could mediate this effect, we monitored the level of hemocytes in adult flies expressing *SpRIP2* and *BiP* + *SpRIP2* under the control of two ubiquitous *GAL4* drivers. *Hml* expression quantification revealed a decrease in the number of hemocytes in these flies similar to the decrease observed upon *Spiroplasma* infection (Fig. 4c and Additional file 5: Figure S5). These results suggest that *Sp*RIPs cause hemocytes death, which in turn could contribute to aging and premature death of flies.

### *SpRIPs* ectopic expression causes embryo mortality revealing higher in male embryos compare to female ones

**Fig. 5:**
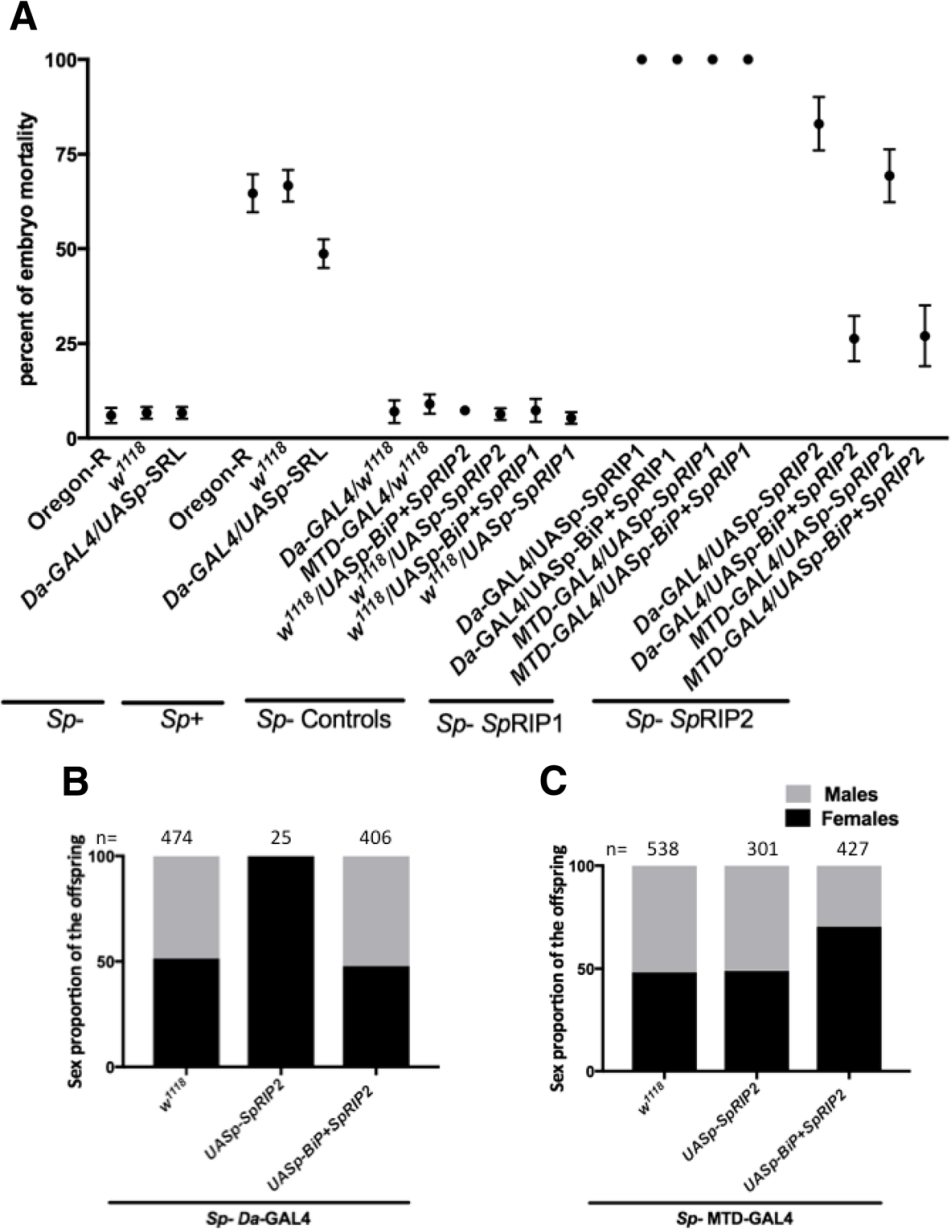
**a** Effect of *SpRIP* expression on embryo mortality. **b** Sex ratio of uninfected flies expressing *UASp-SpRIP2* under *Da-GAL4* control (ubiquitous). **c** Sex ratio of uninfected flies expressing *UASp-SpRIP2* under *MTD-GAL4* control (maternal specific). n indicates the number of adult flies counted for the assay. *Sp*- and *Sp +* refer to uninfected or *Spiroplasma*-infected condition respectively

We have shown that RIP activity is particularly high in *Spiroplasma*-infected embryo compared to other developmental stages (Fig. 1c) raising the possibility that *Sp*RIP1 and *Sp*RIP2 could contribute to embryo mortality. To test this possibility, we first monitored the effect of the ectopic expression of *SpRIPs* in uninfected individuals by using either the ubiquitous zygotic *Da*-*GAL4* driver in embryos or the maternal driver *MTD-GAL4*. We monitored embryo mortality as the percent of embryos that do not hatch, which is about 5% in uninfected wild type embryos (Fig. 5a). All uninfected embryos with ectopic expression of *SpRIP1* or *BiP* + *SpRIP1* die, reflecting the high toxicity of *Sp*RIP1. However, the expression of *UASp-SpRIP2* kills about 70% of the embryos (Dunnett’s multiple comparison test against uninfected w1118 p** < 0.0074). Interestingly, over-expression of *UASp-BiP + SpRIP2* shows a lower toxicity with a mortality rate up to 30% (Dunnett’s multiple comparison test against uninfected w1118 p*** < 0.0001) (Fig. 5a). We hypothesized that the secretion of the toxin out of the embryo’s cells reduces its toxicity. To reinforce the hypothesis that RIP activity is indeed responsible for embryo death, we measured RIP activity during embryogenesis for each construct. We observed a correlation between the level of RIP activity and the mortality (Pearson’s correlation test p*** < 0.001) (Additional file 6: Figure S6), suggesting that the mortality indeed results from RIP activity.

To further test the possible implication of *Sp*RIPs in embryo mortality, we took advantage of the *UASp-SRL* construct by analyzing whether buffering RIP activity with additional SRL target could rescue *Spiroplasma*-infected embryos from dying. We first observed that embryonic lethality reaches about 65% in *Spiroplasma-*infected flies, well above the expected 50% if males only were dying. This suggests that not only does Spiroplasma kill males, but also a small fraction of the female progeny, roughly estimated at 12.5% (Dunnett’s multiple comparison test p*** < 0.0001). Interestingly, ectopic expression of SRL slightly decreases mortality of infected embryos by 10% (Dunnett’s multiple comparison test p*** < 0.0001) (Fig. 5a). The sex ratio of the surviving embryo was still 100% females, suggesting that the buffering of RIP activity by the *UASp-SRL* construct is sufficient to rescue females but not males (Fig. 5a).

While the sex-ratio of uninfected hatching flies is of 50% females and 50% males (Fig. 5b, c and Additional file 7: Figure S7), the sex-ratio of uninfected *D. melanogaster* hatching flies from embryos expressing *SpRIP2* were biased toward female. It ranged from 70% when the construct is under the control of the maternal driver *MTD* (Fisher’s exact test p*** < 0.001) to 100% when the ubiquitous driver *Da*-*GAL4* was used (Fisher’s exact test p*** < 0.001) (Fig. 5b and c), suggesting that males are more sensitive to RIP activity.

## Discussion

*S. poulsonii* protects its host against macro-parasites such as nematodes and parasitoid wasps and RIP toxins has been shown to play a major role in this protection [30, 31]. In this study, we provide evidence that *Spiroplasma* RIPs could affect symbiosis beyond their implication in endosymbiont-mediated protection by harming the host and contributing to lifespan shortening and embryo mortality.

We show that only two out of the five putative RIP genes contained in *Spiroplasma* genome are expressed all along the life cycle of *Drosophila* with peaks during embryogenesis and late adulthood. As *S. poulsonii* titer increases with time [19], we hypothesize that the peak in old adults is a consequence of the high density of *Spiroplasma* in the host hemolymph, rather than a change in the gene expression in the bacteria. Infected embryos also reveal particularly high RIP activity, likely due the transmission of RIPs and of already depurinated ribosomes from the mother. Our over-expression studies were carried out with the ubiquitous *Da*-*GAL4* driver as it revealed the closest expression level to natural infection. Such studies confirm that *SpRIP1* and *SpRIP2* target the 28S rRNA of its host as previously shown [30]. Transgenic fly lines expressing *Sp*RIP1 display a higher mortality rate for embryos and larva than those expressing *Sp*RIP2, which may result from a higher RIP1 transgene expression (Additional file 3: Figure S3). Last, the addition of a *Drosophila* secretion signal to the protein sequence tends to reduce its toxicity, which is consistent with *Sp*RIPs targeting 28S rRNA within the cells.

Previous studies have shown that *Spiroplasma* shortens the fly lifespan but the underlying mechanism was unknown, although the synthesis of cardiolipins by the bacteria has been proposed as a cause [19]. Our results suggest that *Spiroplasma* RIPs contribute to the premature death of infected flies. According to this model, the increasing *Spiroplasma* titer in aging flies is accompanied by an increase of *Sp*RIP release in the fly hemolymph, which eventually damages the host tissues. Ectopic expression of *SpRIPs* within cells can be more toxic than natural bacterial expression, as ribosomes are then more exposed to the toxin. This situation is however happening naturally only during the first 3 h of embryogenesis (before the cellularization), after which *Spiroplasma* is extracellular. The addition of a *Drosophila* secretion signal to the toxin thus better mimics the natural situation. However, *Drosophila* life span remained shortened even upon *SpRIP* secretion, suggesting that the toxin is able to enter the cells and depurinate ribosomes efficiently.

Similarly to the phenotype of *Spiroplasma*-infected flies, we show that over-expression of *SpRIP1* and *SpRIP2* are associated with an increase in embryo lethality, a shorter lifespan and a decrease in hemocytes number. While *Sp*RIPs contribute to the protection against *Drosophila*’s parasites, our study suggests that these toxins have also a strong detrimental effect in the host with a tangible impact in late adulthood. This suggests that *Spiroplasma* has not developed any mechanism to shut down RIP expression at the adult stage or in absence or parasite infections. Maintaining a constitutive *Sp*RIP production could be a way to react as quickly as possible to parasite infections at a low cost for the host. It is indeed likely that the fitness cost associated with lifespan reduction in *Drosophila* is minimal, as most eggs are laid during the first 2 weeks [48].

## Conclusion

Insect endosymbioses encompass a continuum of interactions ranging from mutualism to parasitism. In some cases however, assessing the beneficial or detrimental nature of the interaction for the host can be delicate. The *Spiroplasma*/*Drosophila* symbiosis is a prime example of such versatile ecological outcome: the bacteria protects its host against widespread parasites, conferring a major ecological benefit, but also kills male progeny and drastically reduces the adults lifespan, reflecting a pathogenic interaction. *Sp*RIPs are involved in these two different faces of *Spiroplasma* endosymbiosis. They are directly involved in host protection against parasites, but can also cause strong damage to the host in absence of parasite infection, making them the first described endosymbiont-encoded toxins to directly harm its adult host.

## Methods

### Fly stocks and handling

Infected lines were generated in 2011 by injection of *Spiroplasma*-infected hemolymph in Oregon-R females. [17]. Infected lines have been maintained in the laboratory establishing genetically identical lines of Oregon-R Spiroplasma infected and uninfected. Hemocytes were observed on 4 weeks old females *w1118*; *HmlΔGAL-4 > UAS-GFP* [49]*.* For all the experiments, flies were maintained at 25 °C on standard cornmeal medium. Embryos were collected from 5 to 7 days old flies by using cages and yeasted grape juice plates. Lifespan experiments were done as described in [17]. The driver for REP assay (*ey-GAL4*) was obtained from Bloomington stock center (#8221). All experiments have been repeated three independent times.

### RNA, DNA extractions and RT-qPCR

RNA, DNA extractions, and RT-qPCR were performed as described in [17, 19, 28]. Reverse transcription was done using 500 ng of RNA per sample, which was isolated from 3 adult flies, 3 larvae, 3 pupae, or 100 to 300 embryos. *SpRIPs* expression and activity were measured along the whole life cycle by RT-qPCR. Expression for each *SpRIP* was analyzed individually except for *SpRIP3*, *SpRIP4* & *SpRIP5* that were measured with a single pair of primers because of their high sequence identity. RT-qPCR calculations for the expression level of *SpRIPs* was done following the ΔCT method normalizing by *dnaA* expression level. Primers for *dnaA* are described in [17]. RT-qPCR calculations to compare the expression level of *SpRIPs* between the transgenic fly lines and *Spiroplasma*-infected flies was done following the ΔCT method normalizing by *rps17* expression level. Primers for UAS-*SpRIP1* expression are Forward: 5′- CGT AGC AGGTGGTGTTGTTC-3’ Reverse: 5′- GCTTCACCCACATCAGCAAG-3′ (efficiency = 1.81). Primers for UAS-*SpRIP2* expression are Forward: 5′- CGT AGC TCGATACCAGCGTGACCATC-3’ Reverse: 5′- CGTTCTGCAGGTTGTACTCG-3′ (efficiency = 1.94). RIP activity assay was performed as described in [31]. All calculations for RIP activity and hemocyte count have been done following the ΔΔCT method and these figures represent the fold change between the experimental condition samples and the controls which are valued as 1 [50]. Primers for *hml* are: Forward: 5’-GAGCACTGCATACCCCTACC-3’ Reverse: 5’-CCGTGCTGGTTACACTCCTT-3′ (efficiency = 1.88). Gene expression levels were normalized to *rps17*. Figures and statistical results were obtained using GraphPad Prism 7.0b software. All experiments have been repeated three independent times.

### Design and construction of *UAS-SpRIP1* and *UAS-SpRIP2* constructs

*Spiroplasma* has an alternative genetic code and a strong codon bias compared to *Drosophila* [38]*. SpRIP1* and *SpRIP2* gene sequences were codon optimized for insect translation using Geneious v8.1.9. The secretion signal from the sequence of BiP (Hsc70-3) was added at the 5′ end of the RIP genes flanked by two BglII restriction sites. The optimized *BiP-SpRIP1* and *2* were fully synthesized and cloned in a pDONR221 vector for Gateway cloning by Invitrogen GeneArt gene synthesis services. Optimized *SpRIP1* and *2* were obtained from *BiP + SpRIP1* and *2* by digestion of the *BiP* sequence by BglII and re-ligation of the plasmid on itself. The fragment of 28S rRNA was amplified from Oregon-R flies and also cloned in pDONR221. All transgenes were cloned into a *UASp* and a *UASt* vector by Gateway LR reaction and injected in *D. melanogaster* w1118 embryos by Bestgene Inc., Chino Hills, USA.

### Embryo mortality assay

A total of 100 embryos were collected per genotype on grape juice plates 15-20 h after egg laying. After ten more hours, the remaining embryos that did not hatch (dead) were counted. Experiments were done simultaneously with two different drivers, the ubiquitous *Da*-*GAL4*, and the maternal *MTD-GAL4*. All experiments have been repeated three independent times.

## Additional files

Additional file 1: **Figure S1.** *SpRIPs 3,4 and 5* expression level in infected flies along *Drosophila* development stages (One way ANOVA; development stage *p* = 0.9992). (TIF 1384 kb)

Additional file 2: **Figure S2.** Lifespan of infected and uninfected wild types flies (controls for Fig. 3). *Sp*- and *Sp +* refer to uninfected or *Spiroplasma*-infected condition respectively. (TIF 1983 kb)

Additional file 3: **Figure S3.** Expression level of (A) *SpRIP1* and (B) *SpRIP2* in embryos from uninfected, infected and transgenic fly lines expressing UAS-RIP normalized by host *rsp17* transcript level (One way ANOVA; p** = 0.0031 for *SpRIP1* and p** = 0.0049 for *SpRIP2*). (C) Expression level of *SpRIP2* in adults from uninfected, infected and transgenic fly lines expressing UAS-RIP normalized by host *rsp17* transcript level (One way ANOVA; p** < 0.0081). (TIF 5534 kb)

Additional file 4: **Figure S4.** (A) Comparison of RIP activity in *Spiroplasma-*infected embryos with uninfected transgenic embryos expressing *SpRIPS* (One way ANOVA; SpRIP1 p*** = 0.001; One way ANOVA; SpRIP2 p** = 0.0021). (B) Intact 28S rRNA quantification in infected embryos and uninfected transgenic fly lines (C) Comparison of RIP activity in *Spiroplasma-*infected adult flies with uninfected transgenic adult fly (One way ANOVA; SpRIP2 p*** = 0.001) (D) Intact 28S rRNA quantification in infected adult flies and uninfected transgenic adult flies. (TIF 6378 kb)

Additional file 5: **Figure S5.** *hml* transcription level in lines expressing *UAS-SpRIP2* and *BiP + SpRIP2* under *actin*-*GAL4* control. Expression of both constructs also leads to a decrease in hemocyte number (Dunnett’s multiple comparisons test p*** < 0.0001). Controls are normalized as 1. (TIF 1147 kb)

Additional file 6: **Figure S6.** Correlation between RIP activity in embryos aged 0 to 24 h after egg laying and embryo mortality (Pearson’s test *p* < 0.0001). RIP activity in infected wild types was normalized by uninfected samples. Transgenic fly lines were normalized by *Da*-GAL4/*w1118*. Controls are normalized as 1. (TIF 3499 kb)

Additional file 7: **Figure S7.** Sex ratio of the control fly lines for Fig. 5. *Sp*- and *Sp +* refer to uninfected or *Spiroplasma*-infected condition respectively. (TIF 4019 kb)

## Abbreviations

ARP: Adhesion Related Proteins
qPCR: Quantitative Polymerase Chain Reaction
REP: Rough Eye Phenotypes
RIP: Ribosome Inactivating Protein
RT: Reverse Transcription
*Spaid*: *S. poulsonii androcidin*
*Sp*RIP: *S. poulsonii* Ribosome Inactivating Protein
