# Supplementary figures and images for: Functional analysis of RIP toxins from the Drosophila endosymbiont Spiroplasma poulsonii
Source: BMC Microbiol. 2019 Feb 20;19:46. doi: 10.1186/s12866-019-1410-1 (PMC6383259; doi:10.1186/s12866-019-1410-1)

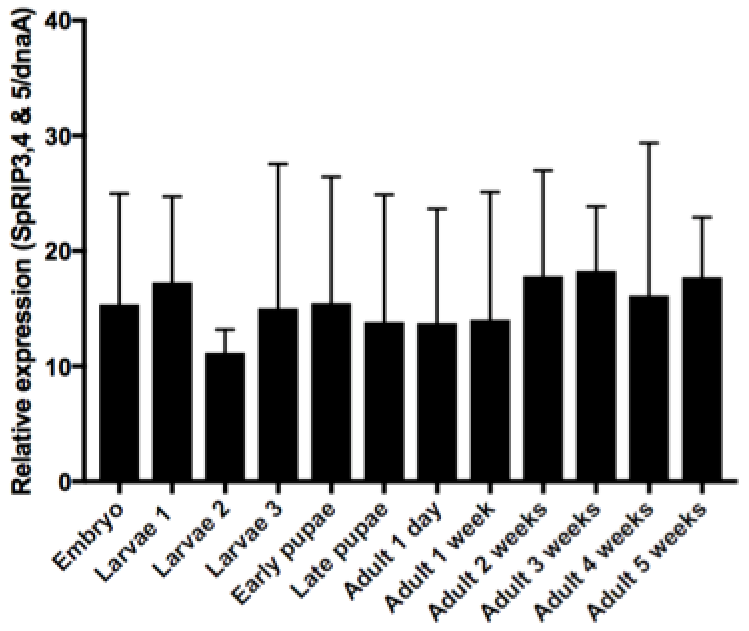

Supplement: Supplementary file 1 — Figure S1. SpRIPs 3,4 and 5 expression level in infected flies along Drosophila development stages (One way ANOVA; development stage p = 0.9992). (TIF 1384 kb) [file 12866_2019_1410_MOESM1_ESM.tif]

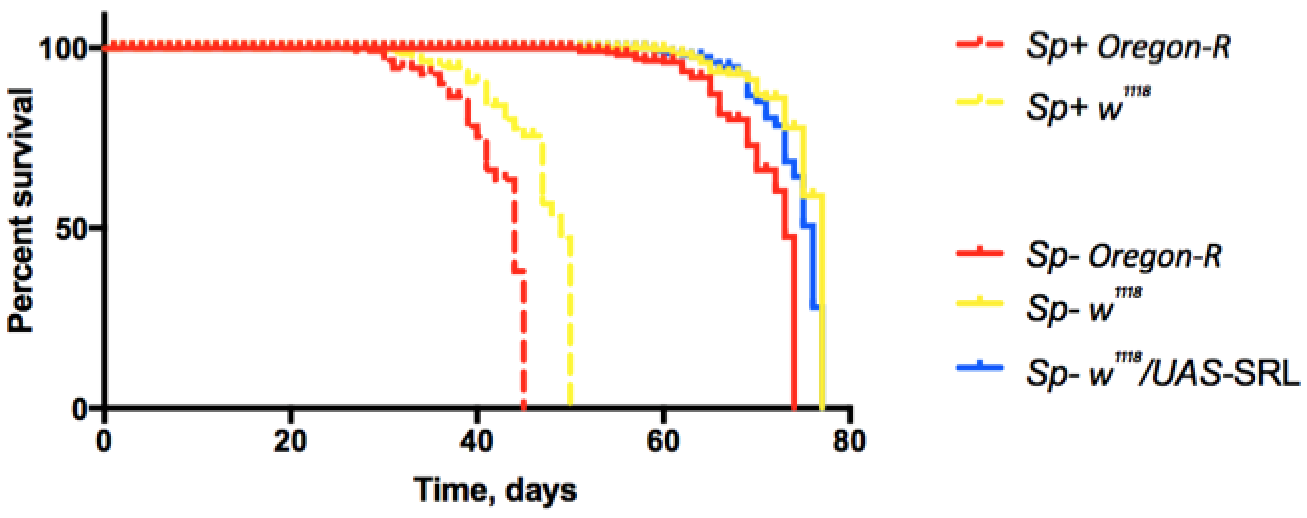

Supplement: Supplementary file 2 — Figure S2. Lifespan of infected and uninfected wild types flies (controls for Fig. 3). Sp- and Sp + refer to uninfected or Spiroplasma-infected condition respectively. (TIF 1983 kb) [file 12866_2019_1410_MOESM2_ESM.tif]

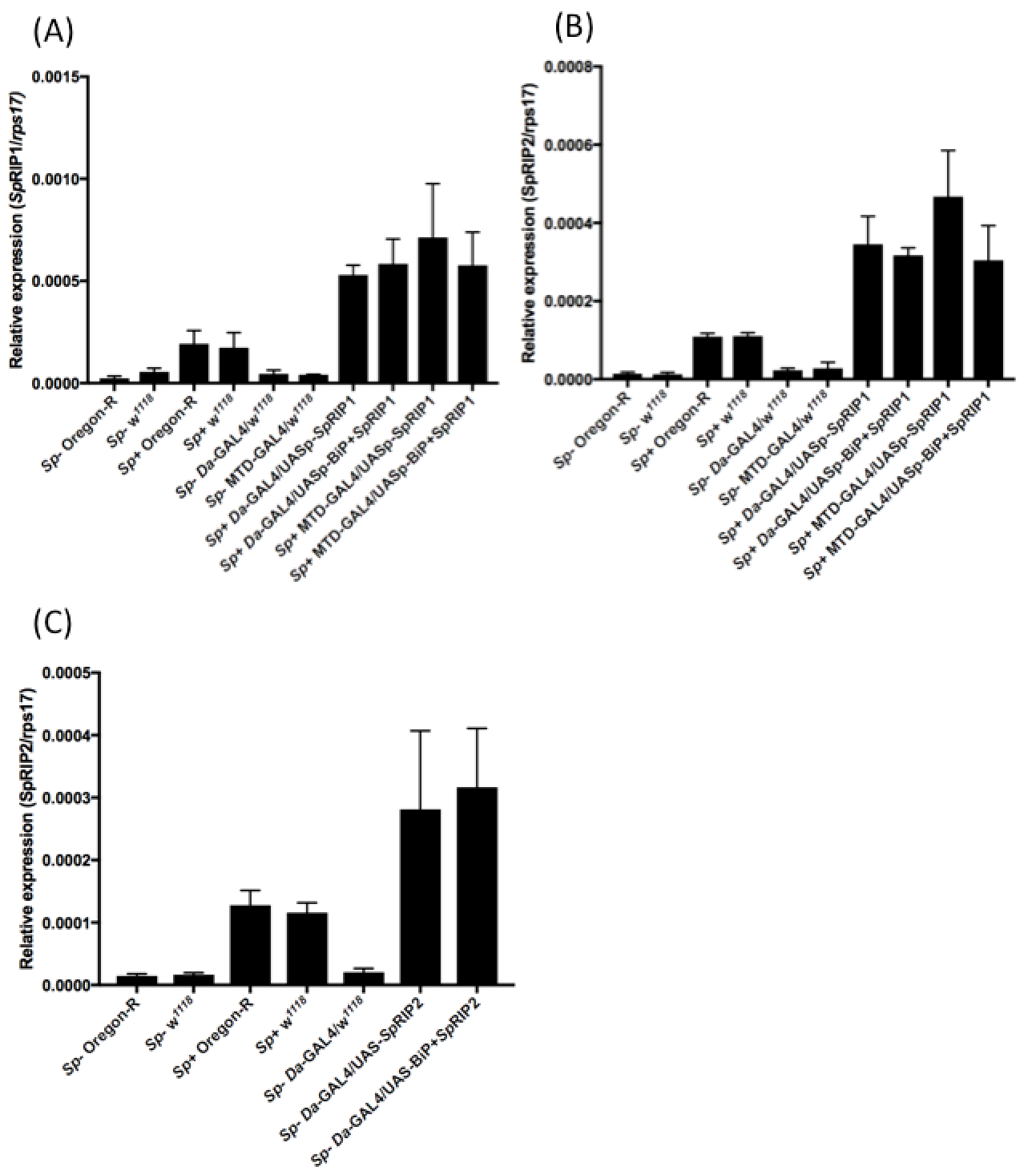

Supplement: Supplementary file 3 — Figure S3. Expression level of (A) SpRIP1 and (B) SpRIP2 in embryos from uninfected, infected and transgenic fly lines expressing UAS-RIP normalized by host rsp17 transcript level (One way ANOVA; p** = 0.0031 for SpRIP1 and p** = 0.0049 for SpRIP2). (C) Expression level of SpRIP2 in adults from uninfected, infected and transgenic fly lines expressing UAS-RIP normalized by host rsp17 transcript level (One way ANOVA; p** < 0.0081). (TIF 5534 kb) [file 12866_2019_1410_MOESM3_ESM.tif]

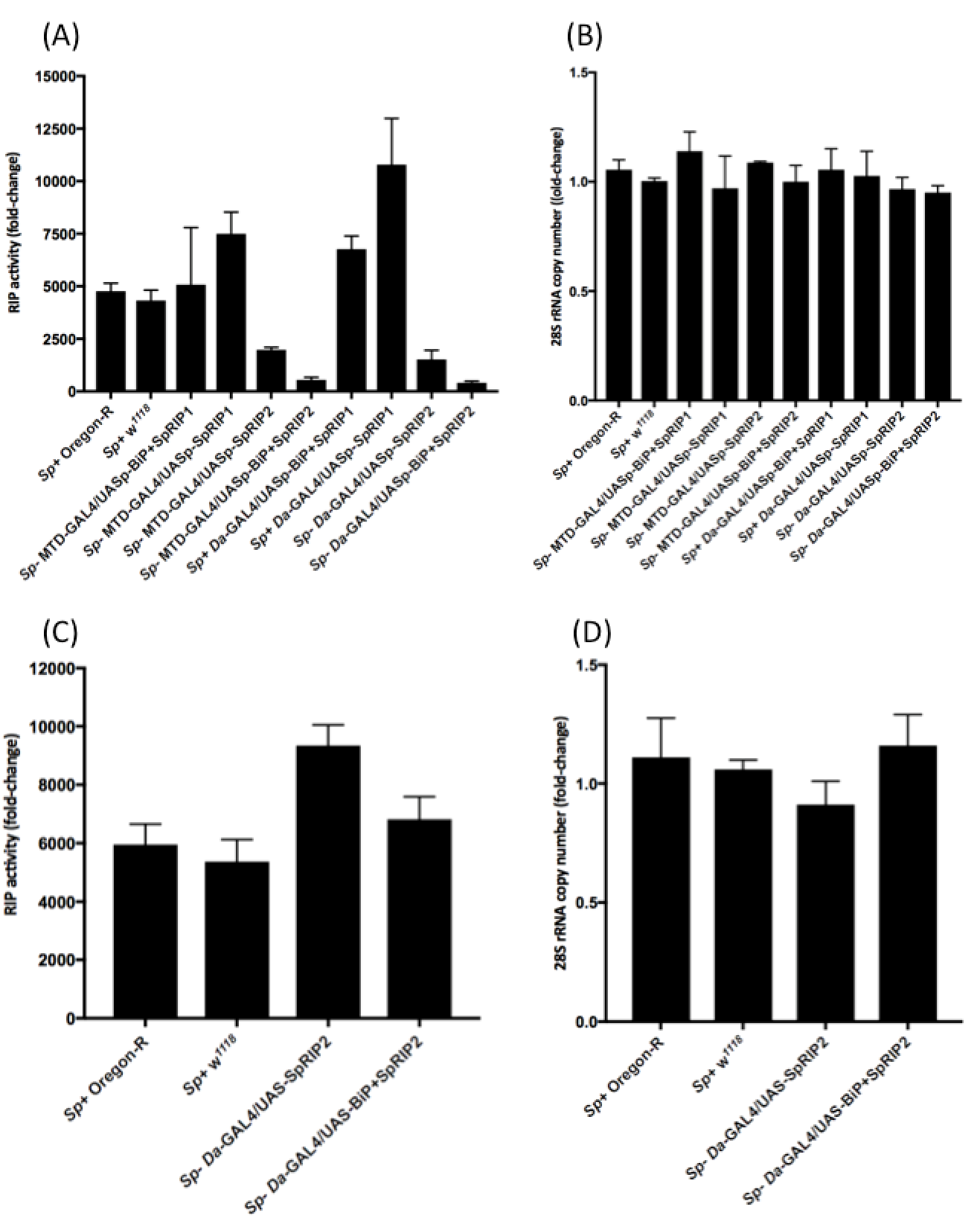

Supplement: Supplementary file 4 — Figure S4. (A) Comparison of RIP activity in Spiroplasma-infected embryos with uninfected transgenic embryos expressing SpRIPS (One way ANOVA; SpRIP1 p*** = 0.001; One way ANOVA; SpRIP2 p** = 0.0021). (B) Intact 28S rRNA quantification in infected embryos and uninfected transgenic fly lines (C) Comparison of RIP activity in Spiroplasma-infected adult flies with uninfected transgenic adult fly (One way ANOVA; SpRIP2 p*** = 0.001) (D) Intact 28S rRNA quantification in infected adult flies and uninfected transgenic adult flies. (TIF 6378 kb) [file 12866_2019_1410_MOESM4_ESM.tif]

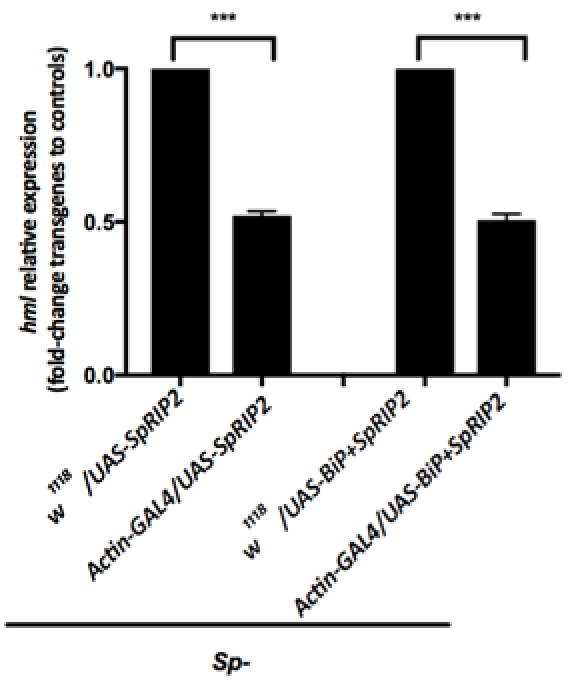

Supplement: Supplementary file 5 — Figure S5. hml transcription level in lines expressing UAS-SpRIP2 and BiP + SpRIP2 under actin-GAL4 control. Expression of both constructs also leads to a decrease in hemocyte number (Dunnett’s multiple comparisons test p*** < 0.0001). Controls are normalized as 1. (TIF 1147 kb) [file 12866_2019_1410_MOESM5_ESM.tif]

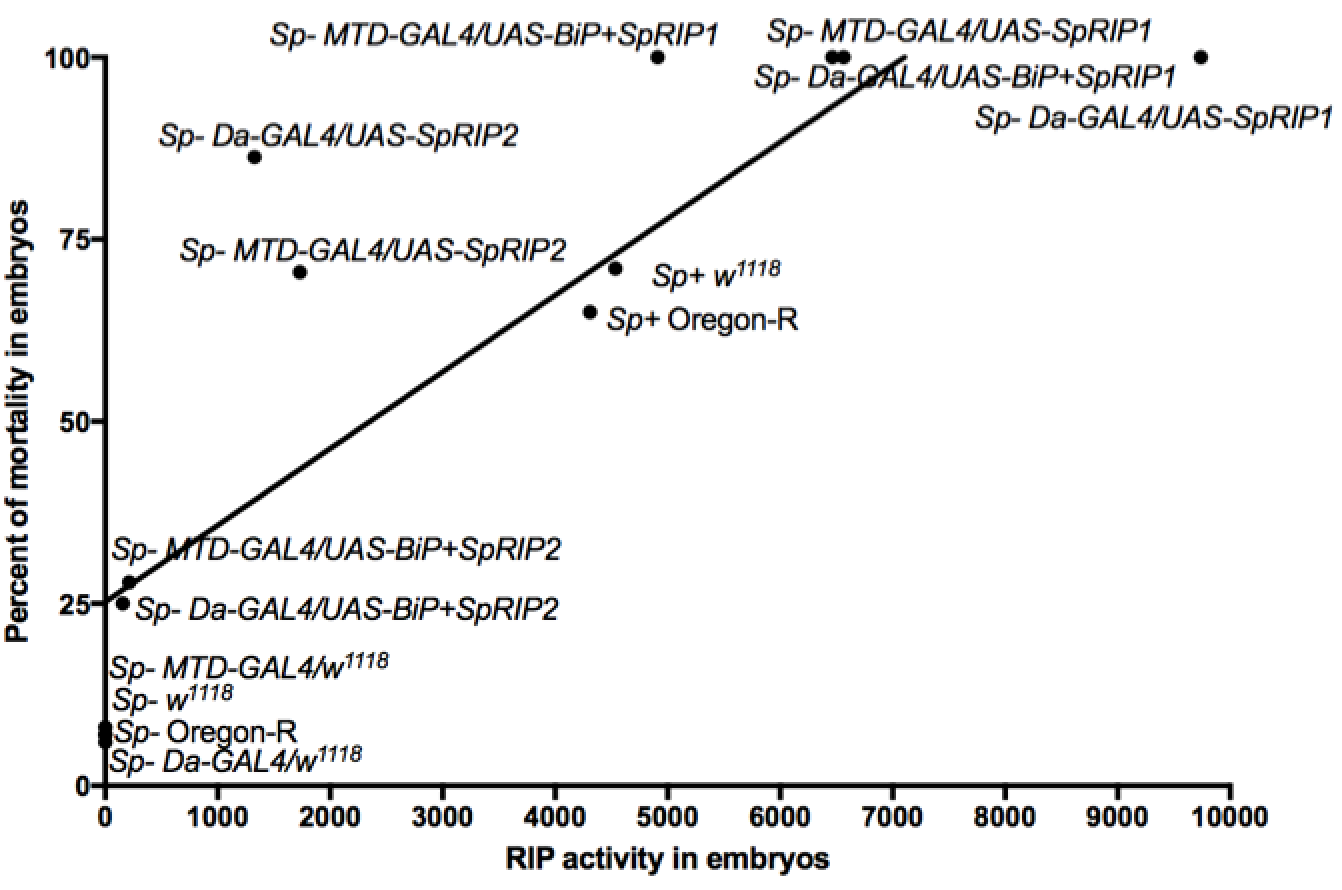

Supplement: Supplementary file 6 — Figure S6. Correlation between RIP activity in embryos aged 0 to 24 h after egg laying and embryo mortality (Pearson’s test p < 0.0001). RIP activity in infected wild types was normalized by uninfected samples. Transgenic fly lines were normalized by Da-GAL4/w1118. Controls are normalized as 1. (TIF 3499 kb) [file 12866_2019_1410_MOESM6_ESM.tif]

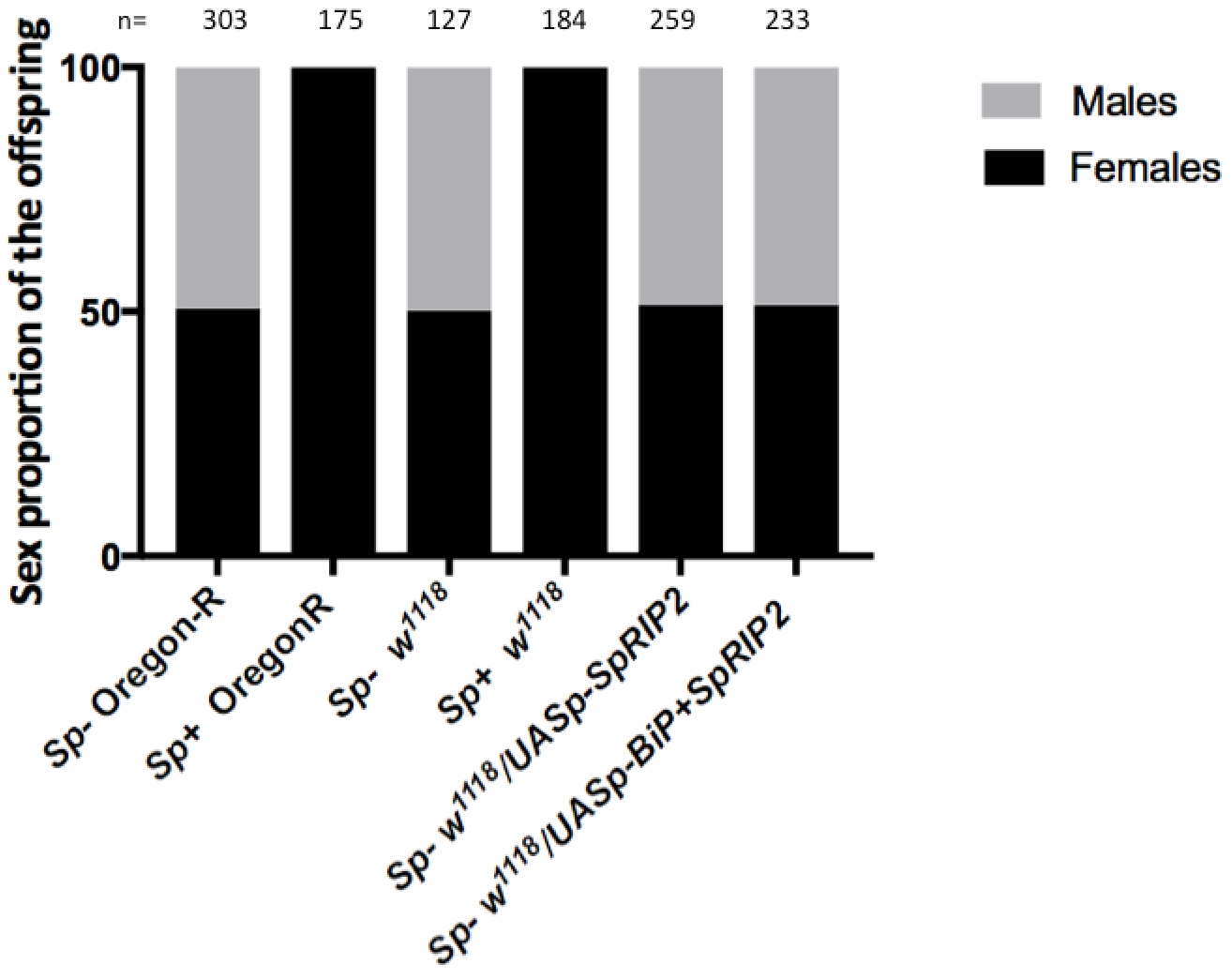

Supplement: Supplementary file 7 — Figure S7. Sex ratio of the control fly lines for Fig. 5. Sp- and Sp + refer to uninfected or Spiroplasma-infected condition respectively. (TIF 4019 kb) [file 12866_2019_1410_MOESM7_ESM.tif]
